# Mechanisms of vascular smooth muscle cell investment and phenotypic diversification in vascular diseases

**DOI:** 10.1042/BST20210138

**Published:** 2021-09-08

**Authors:** Matthew D. Worssam, Helle F. Jørgensen

**Affiliations:** Cardiovascular Medicine Division, University of Cambridge, Cambridge, U.K.

**Keywords:** cardiovascular disease, cell plasticity, lineage tracing, single cell RNA sequencing, vascular smooth muscle

## Abstract

In contrast with the heart, the adult mammalian vasculature retains significant remodelling capacity, dysregulation of which is implicated in disease development. In particular, vascular smooth muscle cells (VSMCs) play major roles in the pathological vascular remodelling characteristic of atherosclerosis, restenosis, aneurysm and pulmonary arterial hypertension. Clonal lineage tracing revealed that the VSMC-contribution to disease results from the hyperproliferation of few pre-existing medial cells and suggested that VSMC-derived cells from the same clone can adopt diverse phenotypes. Studies harnessing the powerful combination of lineage tracing and single-cell transcriptomics have delineated the substantial diversity of VSMC-derived cells in vascular lesions, which are proposed to have both beneficial and detrimental effects on disease severity. Computational analyses further suggest that the pathway from contractile VSMCs in healthy arteries to phenotypically distinct lesional cells consists of multiple, potentially regulatable, steps. A better understanding of how individual steps are controlled could reveal effective therapeutic strategies to minimise VSMC functions that drive pathology whilst maintaining or enhancing their beneficial roles. Here we review current knowledge of VSMC plasticity and highlight important questions that should be addressed to understand how specific stages of VSMC investment and phenotypic diversification are controlled. Implications for developing therapeutic strategies in pathological vascular remodelling are discussed and we explore how cutting-edge approaches could be used to elucidate the molecular mechanisms underlying VSMC regulation.

## Introduction to VSMC plasticity

Vascular smooth muscle cells (VSMCs) reside in the medial layer of major blood vessels in mammals. They are generally quiescent in healthy, adult vessels, where their contractility controls vascular tone and blood distribution. Upon injury and inflammation, VSMCs undergo ‘phenotypic switching’, characterised by down-regulation of contractility-associated markers (e.g. MYH11, ACTA2), increased synthetic capacity for extracellular matrix (ECM) components, induction of proliferation, and enhanced migration [1]. The contribution of VSMCs has historically been considered net-beneficial for some vascular diseases, e.g. in atherosclerosis, through plaque stabilisation by fibrous cap thickening [2,3]. In contrast, the effect of VSMCs is deemed net-detrimental in the vessel-narrowing and occlusion of in-stent restenosis and PAH [4,5]. However, the ability to pinpoint the roles of VSMCs in disease was historically limited by the loss of classical contractile markers in phenotypically switched VSMCs, impeding their identification in diseased tissues [2]. Use of the tamoxifen-inducible CreER^T2^ recombinase under the control of VSMC-specific promoters (e.g. *Tagln* and *Myh11*) has enabled robust lineage tracing of existing differentiated VSMCs and their progeny through the progression of vascular disease, regardless of subsequent fate changes [6]. Experiments using this technique showed that existing VSMCs in the arterial wall are the major constituents of remodelled regions in atherosclerosis [7–11], vascular injury [7,12,13], aneurysm [14,15] and PAH [16–18]. Genetic lineage-labelling also revealed that VSMC plasticity in disease exceeded what was previously appreciated (reviewed by Liu and Gomez) [19]. For example, in addition to contributing to cap-stabilising αSMA+ cells, many VSMCs adopt plaque-destabilising inflammatory and calcifying states in atherosclerotic lesions. The extent of VSMC diversification in vascular diseases has recently been mapped using single-cell RNA-sequencing (scRNA-seq) in VSMC-lineage traced vascular disease models [14,20–24]. Insights from VSMC-lineage tracing and scRNA-seq have suggested that the pathway from contractile VSMCs in healthy arteries to phenotypically distinct lesional cells consists of multiple steps that could be controlled independently. For example, activation of VSMC proliferation and cell migration into the lesion may be governed by distinct molecular mechanisms [7,17,25]. To realise the therapeutic potential of targeting VSMCs in vascular pathogenesis, several key questions therefore should be addressed. (1) What are the stages of VSMC investment and differentiation in vascular pathogenesis? (2) What are the molecular mechanisms that govern specific stages? (3) How might intervention at one stage affect other stages? Here, we will discuss recent insights, primarily gained from atherosclerosis, a well-studied cardiovascular disease with pathogenic VSMC contribution.

## Stages of VSMC lesion investment

**Figure 1.**
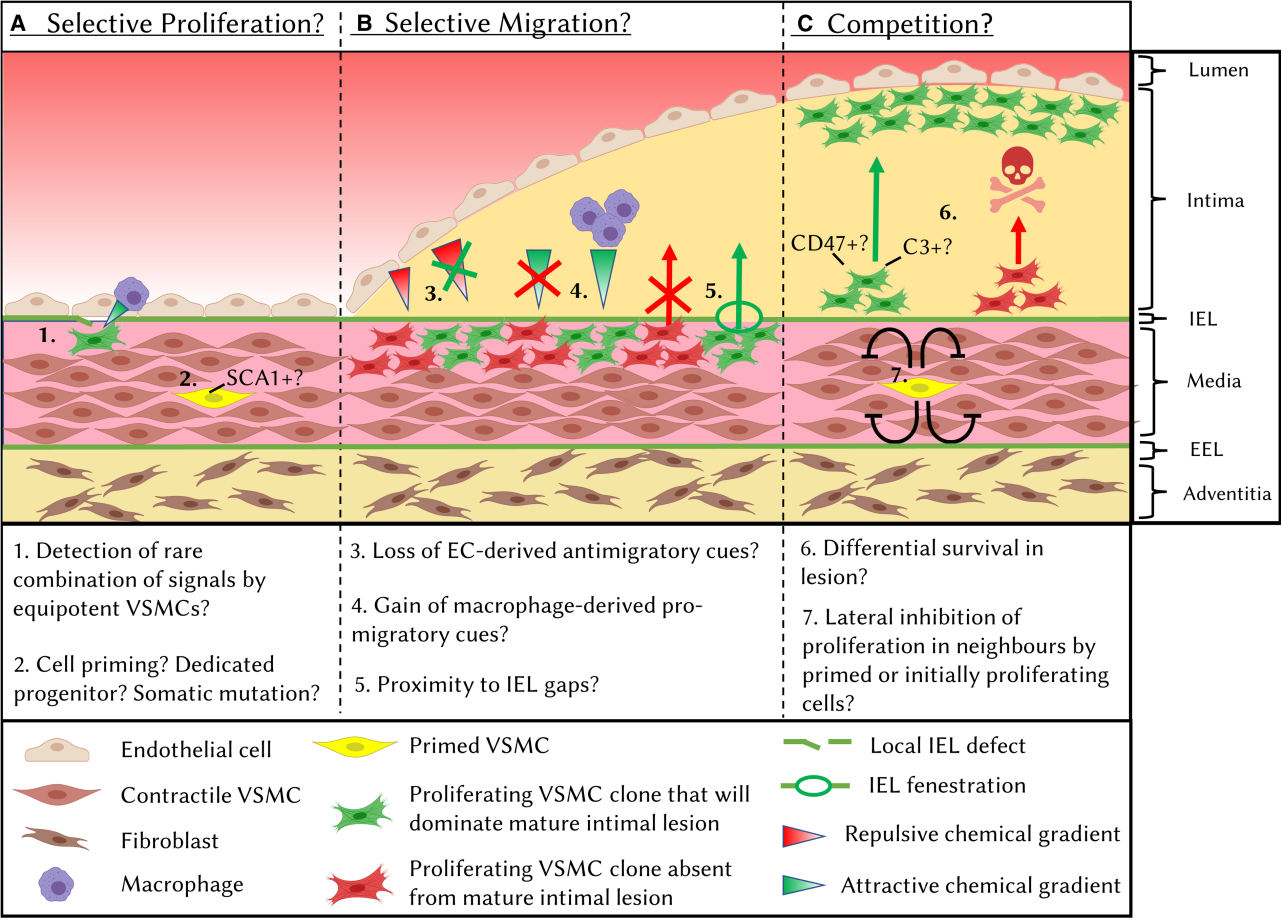
Mechanisms that may underlie oligoclonal VSMC lesion contribution. Mature vascular lesions show contribution from few VSMC clones. Oligoclonality could arise from selective mechanisms at the level of activation of cell proliferation (**A**) or intimal invasion of expanding VSMC clones (**B**) or be due to competition for survival (**C**). Differential success between VSMC clones at each stage could be due to stochastic differences in environmental signals or to cell-intrinsic differences between clones. These mechanisms are not mutually exclusive. IEL: internal elastic lamina, EEL: external elastic lamina.

Using multicolour lineage tracing systems, where cells stochastically express one of multiple differently coloured reporter proteins that are inherited by progeny, it was shown that the extensive VSMC contribution to vascular remodelling arises from the clonal expansion of few pre-existing cells in atherosclerosis [7,8,10,23,26], acute vascular injury [7,27], aneurysm [14,15] and PAH [16,17]. The observation of VSMC clonal expansion in multiple diseases suggests that selective cell expansion is a programmed feature of adult VSMCs, in contrast with the widespread proliferation and extensive mixing of VSMC clones during embryonic development [10,28]. It remains controversial whether VSMC oligoclonality in mature vascular lesions is due to selective intimal investment of few VSMC clones which all show high survival rates in the lesion (Figure 1A,B) or results from a competition for survival between many clones (Figure 1C) [29]. Moreover, selective intimal investment could in turn result from the selective activation of proliferation in few VSMCs (Figure 1A), the selective capacity for migration into the lesion of few expanding medial clones (Figure 1B), or a combination of both. Here we discuss the current evidence for selective and competitive processes during VSMC-lesion investment.

### Selective intimal investment

#### A primed/progenitor VSMC population in the systemic vasculature?

Selective activation of VSMC proliferation could arise from the stochastic detection of a rare combination of signals amongst equipotent VSMC clones. However, evidence from PAH models suggests that the clonal expansion of VSMCs into previously non-muscularised pulmonary arterioles [16,17] and into the intima of larger pulmonary arterioles [18] arises from the selective ability of primed cells, marked by PDGFRβ and NOTCH3 respectively, to respond to pathological signals. Although the existence of a primed or progenitor population in the systemic vasculature has not been definitively proven, accumulating evidence supports this hypothesis (Figure 1A). In particular, scRNA-seq has revealed extensive heterogeneity of VSMCs in healthy vessels, including a small number of VSMCs in the mouse aorta expressing the transcript *Ly6a* which encodes stem cell antigen-1 (SCA1) [21]. Many SCA1+ VSMCs showed significant transcriptional differences to the bulk medial VSMC population, including reduced levels of contractile marker transcripts and expression of activation-associated genes. Notably, an increased proportion of VSMC-derived cells express SCA1+ in the plaque [21,24,30], in aneurysm [14] and after acute injury [21], which could be accounted for by expansion of an original SCA1+ population. However, definitively proving this using lineage tracing of SCA1+ VSMCs from healthy to diseased conditions is technically challenging as (1) SCA1 is also expressed in adventitial and endothelial cells, necessitating the use of a dual-lineage tracing system [20,31] and (2) SCA1+ VSMCs express the *Ly6a* transcript at levels likely too low to induce Cre-mediated recombination [31,32].

Meta-analysis of scRNA-seq datasets generated from VSMC-lineage traced plaques revealed a continuum in transcriptional profiles between contractile and modulated *Ly6a+/*SCA1+ ‘SEM' (stem cell, endothelial, monocyte [22]) cells [30]. This is inconsistent with a distinct transcriptional response to atherogenic stimuli of a dedicated progenitor population compared with the bulk VSMC population. Thus, whilst the presence of SCA1+ VSMCs in the healthy vessel does not preclude the existence of other primed VSMC populations, it is unlikely that a true dedicated progenitor exists. In line with this idea, SCA1-induction in cultured VSMCs that were initially SCA1- [21] suggests that this state is not reserved for specialised cells but could represent dynamic cell priming.

Targeting a potential primed VSMC state would be an efficient method to prevent acute VSMC proliferation, particularly for the prevention of restenosis following vessel injury. However, if ablation permanently removes primed VSMCs, physiological repair of minor injuries could be severely disrupted. Moreover, targeting a primed cell state may not be desirable in atherosclerosis as VSMC-contribution is believed to have net-beneficial roles on atherosclerotic plaque stability [24]. In the context of neovascularisation post-ischaemia, promoting VSMC priming, and thus enhancing injury-induced VSMC proliferation, could represent a viable therapeutic strategy to protect nascent endothelial vessels.

#### Selective VSMC migration

The local environment may significantly impact the resulting clonality of vascular lesions (Figure 1B). For example, VSMC lesion invasion could depend on proximity to fenestrations in the internal elastic lamina (IEL), as confocal microscopy analysis showed these allow migration of aortic VSMCs into the intimal space [25]. Interestingly, the internal mammary artery, widely used for bypass grafting due to its greatly reduced restenosis rate compared with other vessels, has a much lower density of IEL fenestrations compared with other vessels [33], further suggesting that *trans*-IEL migration may represent a rate-limiting step. VSMCs have also been reported to invade the atherosclerotic plaque through pathological IEL fractures, the frequency of which in humans correlates with plaque size [34–36] and possession of the ε4 allele of *APOE* [37]. In response to acute injury, invasion of the neointima is restricted to expanding VSMCs clones [7], suggesting that a combination of the low frequencies of proliferating VSMCs and proximity to IEL fenestrations/fractures may underlie rare VSMC intimal migration. Furthermore, the ability of VSMC clones to invade the intima may be deterministic. Clones with increased proliferative and migratory abilities would be able to ‘find' fenestrations more frequently, whilst those with higher metalloproteinase activity might be able to generate IEL fractures more readily. As evidence of this idea, in muscularised pulmonary arterioles, NOTCH3+ VSMCs were shown to have an intrinsic proficiency to invade the neointima [18].

The control of VSMC intimal invasion is also influenced by cues from other cell types. Lu et al. [25] identified that endothelial cell (EC)-specific loss of *Mef2c*, an event associated with atherogenic disturbed bloodflow, was sufficient to remove inhibition of VSMC migration into the intima, possibly via down-regulation of the repulsive cue *Sema3d*. Furthermore, macrophage-specific knockout of integrin-β3 (*Itgb3*) resulted in increased VSMC clonality in the plaque, revealing the importance of VSMC-macrophage crosstalk in VSMC intimal invasion [10].

### Competition mechanisms

The small number of VSMC clones observed in mature lesions [7,10,23,26] appears at odds with experiments that detected DNA synthesis in many medial cells following acute vascular injury [38–41] and early atherogenesis [42], which suggested widespread activation of proliferation. However, these studies did not use VSMC lineage tracing, and therefore it is likely that non-VSMCs, such as infiltrating immune cells, contributed to the overall proliferation index of cells in the media. Alternatively, many VSMCs could exit quiescence, but subsequently be lost in a clonal competition (Figure 1C). Interestingly, VSMC-derived clones in the core of atherosclerotic plaques have been shown to express the ‘don't-eat-me' marker CD47 [23,43]. Differential expression of CD47 between VSMC clones was proposed to cause differential survival in the plaque and thus account for the dominance of few clones [23]. Notably, treatment with anti-CD47 antibodies increased the number of VSMC clones in the plaque and reduced plaque size without affecting overall VSMC-investment [23]. VSMC-specific *Cd47* deletion could further identify whether the effects of anti-CD47 treatment are due to loss of competitive advantage between VSMC clones or result from actions on CD47-expressing professional efferocytotic cells.

Finally, cell competition could occur at a pre-proliferative stage, where cells that initially activate proliferation, through priming or stochastic mechanisms, prevent surrounding cells from exiting quiescence. Examples of such lateral inhibition mechanisms are found in development, often mediated by Notch-Delta signalling [44].

## Phenotypic diversification of VSMC-derived cells in vascular disease

VSMC lesion contribution has historically been significantly underestimated due to the loss of classical contractile VSMC marker gene expression in a large fraction of VSMC-derived plaque cells, demonstrated by genetic lineage labelling studies [7,9,11,26]. Remarkably, a high proportion of αSMA^−^ VSMC-derived cells stained positively for markers of a wide range of cell types including mesenchymal stem cells (SCA1+) [11,21], myofibroblasts (PDGFRB+) [10,11], osteocytes/chondrocytes (SOX9+, RUNX2+) [45,46], and macrophage/foam cells (CD68+/Oil Red O+) [7,8,10,11,21,23]. Evidence that VSMC diversification also occurs in human atherosclerotic lesions includes detection of cells expressing the macrophage marker CD68 and either αSMA [47] or an epigenetic mark specific to previously contractile VSMCs [9]. Finally, in aneurysm, clonally expanded VSMCs in the false channel and adventitia of ruptured aortae stained positively for the macrophage markers CD68 and LAMP2 [15]. Overall, there is much evidence that VSMC phenotypic diversification occurs during vascular disease progression *in vivo*.

Recent scRNA-seq studies have increased our ability to identify phenotypes adopted by VSMC-derived cell types in vascular disease models beyond staining for a few markers [14,20–24,48]. Comparison of data from several groups provides robust evidence that VSMC-derived atherosclerotic plaque cells contribute significantly to overlapping cell states comprising fibroblast-like and ‘fibrochondrocytic' cells and modulated VSMCs [30,49]. The specific clustering and nomenclature of modulated VSMCs differ between studies. However, the cell populations referred to as ‘fibromyocytes' [24], ‘pioneer cells’ [20] and ‘stem cell, endothelial, monocyte; SEM cells' [22] share expression of a set of markers including *Ly6a*/SCA1, *Lgals3* and *Vcam1* and likely represent similar populations. We henceforth refer to this modulated VSMC state as SEM cells, similar to a recent meta-analysis of this data by Conklin et al. [30]. Modulated VSMC states were also identified in scRNA-seq datasets of human plaques, indicating that they are clinically relevant for human atherogenesis. Integration of scRNA-seq data from mouse and human plaques strongly suggested that these human cell populations represent equivalent states to those observed in lineage-traced mouse models, and overcomes the well-known fact that SCA1 does not have an obvious human orthologue. Conserved markers expressed by modulated VSMCs in both human and mouse atherosclerosis include *FN1*, *TNFRSF11B* and type-I collagens [20,22,24]. In contrast with the robust identification of modulated VSMCs, there were discrepancies in the proportion of VSMC-derived macrophage-like cells between studies, including complete absence of detection in one study [24]. These differences may have arisen from technical issues such as selective loss of VSMC-derived macrophages during sample preparation, or due to biological differences between plaques from different vascular regions.

### What is the lineage relationship between VSMC-derived cells of differing phenotypes?

**Figure 2.**
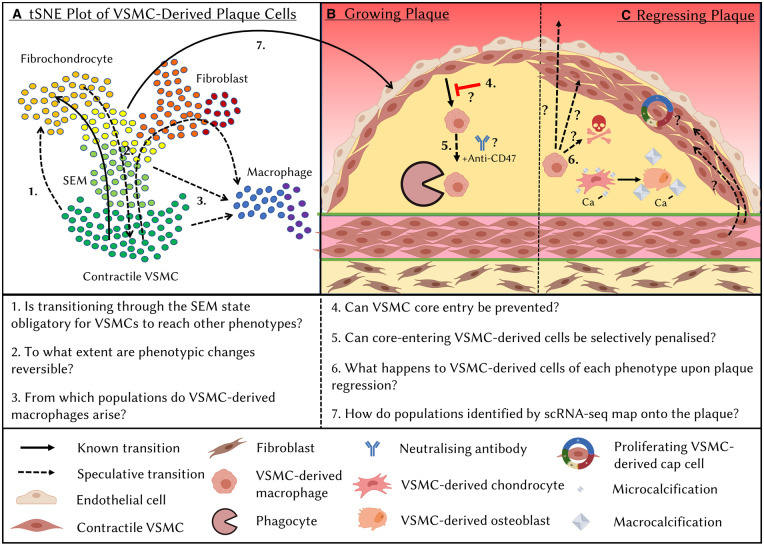
Outstanding questions on VSMC phenotypic diversification in atherosclerotic lesions. Schematic plot illustrating that single-cell RNA-seq (scRNA-seq) technologies have revealed the wide range of transcriptional profiles adopted by VSMC-derived cells in atherosclerotic lesions (**A**). Trajectory inference analysis suggests a transition from contractile VSMCs to a fibrochondrocytic state via an intermediate SEM (stem cell, endothelial, monocyte) state. However, the exact lineage relationships between VSMC-derived cells of different phenotypes remain to be fully determined, particularly the origin of VSMC-derived macrophage cells. Generation of scRNA-seq datasets requires dissociation of the tissue and thus loss of spatial information. Therefore, the impact of spatial localisation within the plaque on VSMC-derived cell fate transitions remains unknown (**B**). For example, would it be possible to inhibit the generation of plaque-destabilising phenotypes by inhibiting VSMC-derived cell entry into the lesion core? Moreover, the reversibility of cell transitions and the ability of VSMC-derived cells in the lesion to convert into other states remains unknown (**A**,**C**). This is of particular interest in the regressing plaque where alterations in the behaviour and/or fates of VSMC-derived cells are likely to have a significant role in plaque stabilising changes (**C**).

Multicolour lineage tracing revealed intrinsic VSMC plasticity by showing that cells from the same VSMC clone can adopt different VSMC-derived plaque phenotypes, but does not inform about lineage relationships [7,26]. Computational trajectory inference analysis of integrated data from several scRNA-seq studies of VSMC-lineage traced plaques suggests that VSMCs first down-regulate contractile marker genes and induce expression of *Ly6a*/SCA1, *Ly6c* and *Lgals3* [30] (Figure 2A). Many cells in the so-called SEM cell cluster express *Ly6a*/SCA1 but lack expression of other MSC markers including *Cd73* and *Cd105*, suggesting that these are a unique cell type specific to VSMC-derived cells [22]. Pan et al. demonstrated that, compared with SCA1-LY6C- cells, SCA1+LY6C+ cells treated with different factors in culture showed increased ability to up-regulate marker gene expression characteristic of multiple cell types including macrophages, fibrochondrocytes and the contractile phenotype [22]. This suggested that SEM cells have increased differentiation potential towards multiple phenotypes. Experimental evidence for SEM cells representing an intermediate state towards VSMC phenotypic diversification *in vivo* comes from elegant dual-lineage tracing using the *Myh11* and *Lgals3* promoters, which showed that 60–80% of VSMC-derived cells in later-stage plaques went through an LGALS3+ state whilst only 20% retained LGALS3 expression [20]. Furthermore, scRNA-seq analysis showed that *Myh11/Lgals3* dual-lineage+ cells contribute to osteogenic, ECM-producing and inflammatory phenotypes, demonstrating significant plasticity *in vivo* [20]. Clonally expanded, VSMC-derived cells that express SCA1, as well as markers of adipocytes, osteoblasts, chondrocytes and macrophages have also been identified in the context of aneurysm [14]. This suggests that mechanisms of initial conversion to an MSC-like state followed by differentiation into one of several phenotypes could be a feature of multiple vascular diseases.

The lineage relationship between VSMC-derived macrophages and other VSMC-derived cell states remains unclear (Figure 2A), particularly as the proportion of the former differs so greatly between scRNA-seq studies. ScRNA-seq analysis of VSMC lineage-traced plaques from multiple groups shows that VSMC-derived macrophage-like cells cluster separately from other VSMC-derived cells [20–22,30]. Interestingly, these studies found only one macrophage marker-expressing cell population, which had contribution from both VSMC-lineage positive and negative cells [20,22,30], suggesting that VSMC-derived macrophage-like cells are similar to their bone marrow-derived counterparts *in vivo*. This is different from observations *in vitro*, where modelling of foam cell formation by stimulation of VSMCs and bone marrow-derived macrophages with modified lipids resulted in substantial transcriptional and functional differences, including in efferocytotic capacity [50]. Conklin et al. [30] identified a trajectory from contractile VSMCs to macrophage-like cells via the SEM state in their meta-analysis, but also showed that the ‘nearest neighbours' in multi-dimensional space of VSMC-derived macrophages were located in several clusters ranging from contractile to fibrochondrocytic, obfuscating the determination of the macrophage-like cell origin. Interestingly, some VSMC-derived macrophages expressed a proliferative signature. Speculatively, differentiation towards a macrophage-like state could represent a second rare clonal event which is followed by rapid clonal expansion of macrophage-like cells. Testing this hypothesis would require clonal lineage tracing of modulated VSMCs. Importantly, a such second clonal event could represent a novel stage for therapeutic targeting.

### Targeting VSMC-derived cells in the lesion core

Lineage-tracing of VSMC-derived cells over a timecourse of aortic root plaque progression suggested that VSMC-derived cells migrate along the fibrous cap before invading the lesion core [10]. Dual-lineage tracing from Alencar et al. [20] corroborated these findings and showed that all plaque-invading VSMC-derived cells in early-stage plaques are derived from LGALS3+ cells, suggesting that SEM cells likely represent cells in the media or early cap. Sequential stages of VSMC cap and core investment, possibly governed by separate mechanisms, would offer the opportunity to inhibit VSMC core entry (Figure 2B). As this might also increase the cellularity of the fibrous cap, it could be an attractive therapeutic strategy. Whilst guidance cues from the local environment may be important for inducing VSMC core entry, changes in cell-intrinsic VSMC migratory abilities might also be important. VSMC-derived cells have been observed spreading across the cap in contiguous sheets [26], perhaps via ‘mesenchymal crawling' on the ECM. Invasion of the core, which has a lower cell density and different ECM protein composition may require a different migration strategy such as ‘ameboid blebbing' used by macrophages [51,52]. Speculatively, differentiation into macrophage-like cells could therefore promote VSMC invasion of the core. An alternative therapeutic strategy would be to selectively penalise the invading cells. To this end, anti-CD47 antibody treatment in mice has been shown to reduce the VSMC-derived core, but not cap, contribution, although the underlying mechanisms were not fully elucidated [23].

### VSMC plasticity in plaque regression

Removal of hypercholesterolemia in animal models of atherosclerosis and human patients causes disease regression. Lipid-lowering only modestly reduces plaque size, yet profoundly impacts plaque morphology, including a significant reduction in the size of the lipid-rich necrotic core and a thickening of the protective fibrous cap [53]. As the major constituent cell type in advanced lesions, VSMC-derived cells could have a significant role in these beneficial changes. Whilst reduction in cholesterol levels may result in expulsion of lipids that could affect VSMC-derived foam cells, it is unclear how other cell type conversions would be directly affected (Figure 2C). The multiple steps involved in generating VSMC-derived plaque cells documented by scRNA-seq suggest that removing the initiating trigger may not simply reverse cell state. In fact, evidence suggests that VSMC-derived osteochondrocytic cells differentiate to mature osteoblasts upon plaque regression, which may change their calcification phenotypes towards production of macrocalcifications, associated with reduced inflammation, rather than plaque-destabilising microcalcifications [54,55]. However, whilst scRNA-seq experiments have been conducted on macrophage-lineage traced regressing plaques [56], the fates of VSMC-derived cell types during plaque regression remain uncharacterised.

## How can we discover mechanisms that control VSMC state and fate?

Computational approaches to extract information from scRNA-seq experiments are currently under rapid development. For example, trajectory inference analysis of scRNA-seq data is a powerful method for identifying dynamic gene regulation across cell state transitions, particularly when multiple algorithms are used to confirm the robustness of inferred trajectories [57]. Based on these inferred trajectories, candidate regulators can be identified as genes which have differential expression along a state transition, between different states or at branchpoints between different trajectories [58,59]. A complementary method for identifying genes driving cell state changes is the reconstruction of gene regulatory networks (GRNs) [23,60]. This involves inference of regulatory interactions between genes associated with a given cell state, for example gene co-expression in scRNA-seq data [61]. Central genes in GRNs represent those whose experimental manipulation is predicted to induce the most significant disruption to the state.

Insight into which transcription factors might drive specific cell states can be gained using algorithms like SCENIC [62], which identify enrichment of transcription factor motifs in the promoters of genes within co-expressed modules. Notably, however, the activity of distal enhancer elements also significantly impacts gene expression and cannot be identified from scRNA-seq alone. As active enhancers are characteristically associated with open chromatin, assays of genome-wide chromatin accessibility, most notably ATAC-seq [63,64], have become a powerful method to identify enhancer regions with cell state-specific accessibility. The association of identified enhancers with genes can be achieved using chromatin conformation capture (3C)-style technologies which allow the identification of 3D chromatin interactions genome-wide. The combination of these technologies has been used effectively to study how genetic variants associated with coronary artery disease (CAD) exert their effects in VSMCs [24,65–67]. Recently, multimodal technologies have allowed RNA-seq and ATAC-seq to be performed simultaneously in single cells [68,69]. Applied in the atherosclerosis context by Örd et al. [70] this allows measurement of the correlation between openness of an enhancer and (1) openness of proximal promoters and (2) gene expression levels, providing strong support for direct regulation.

Whilst advances in these techniques offer significant promise for the elucidation of mechanisms underlying fate transitions, hypotheses generated by bioinformatics approaches always require experimental validation. Studies using scRNA-seq analysis following the VSMC-specific deletion of transcription factors, including *Klf4* [11], *Oct4* [71] and *Tcf21* [24]*,* have confirmed that the distribution of fates adopted by VSMC-derived cells can be altered without completely inhibiting VSMC plaque investment. Thus, biasing VSMCs towards plaque-stabilising phenotypes may be a viable and effective therapeutic strategy. Further insight into which stages of VSMC lesion investment and diversification are controlled by specific regulators (e.g. transcription factors) could be gained through a combination of VSMC-specific knockout and clonal lineage tracing over a timecourse (Figure 1). For example, increased VSMC priming would be evidenced by a greater number of patches of clonally expanding medial VSMCs at early stages, whilst increased intimal invasion or survival capabilities would be evidenced by a higher ratio of neointimal to medial patches.

Notably, the non-uniform distribution of different VSMC-derived cell phenotypes within the lesion [7,26] suggests that signals from the local environment are likely to have an instructive role in VSMC-derived cells’ fate decisions. Several recent analysis tools utilise scRNA-seq data to identify all receptor-ligand pairs expressed by different cell types and thus possible routes of communication between them [72]. These tools could be harnessed to reveal the identity and cellular sources of signals guiding the transition of VSMC-derived cells towards different fates in the lesion. Non-cellular environmental signals may also play instructive roles for VSMC phenotypic diversification including pathological features such as the lipid core or lamina defects. However, the necessity of tissue dissociation for the generation of scRNA-seq datasets removes information on the spatial context. Technologies to fill this gap are under rapid development. Spatial transcriptomics [73], solves this issue by releasing RNA molecules from tissue sections onto slide capture areas with positional barcodes that are added during *in situ* cDNA synthesis, before subsequent high-throughput sequencing. This is a powerful approach, but the application of this technology in the study of individual cells is limited by the current resolution and modest size of the capture area. Complementing spatial transcriptomics, hybridisation-based strategies enable co-detection of multiple genes at the single-cell level. RNAscope [74], which has been relatively widely adopted, is affordable but only permits detection of up to 12 markers. A more recent development, *in situ* sequencing technologies [75] can multiplex signals from over 100 genes but is currently very expensive and lacks comprehensive and user-friendly analysis tools. Undoubtedly, continual improvement of the resolution and cost-effectiveness of spatial methods will usher in unprecedented insights into the control of VSMC state and fate in vascular disease.

## Perspectives

- Vascular smooth muscle cells (VSMCs) play major roles in the pathogenesis of cardiovascular disease, which remains the world's biggest killer.
- The contribution of VSMCs to the pathological vascular remodelling characteristic of cardiovascular diseases arises from the extensive proliferation of few pre-existing, differentiated VSMCs. VSMCs in remodelled regions display remarkable plasticity and can adopt a range of phenotypes that are predicted to have both clinically beneficial and detrimental functions.
- Elucidation of individual steps and underlying mechanisms during the process of VSMC activation (e.g. exit from quiescence, invasion of remodelled vascular regions and phenotypic diversification) will allow for the development of therapeutic strategies to reduce pathological VSMC functions whilst maintaining or enhancing VSMCs’ stabilising roles.

## Open Access Statement

Open access for this article was enabled by the participation of University of Cambridge in an all-inclusive Read & Publish pilot with Portland Press and the Biochemical Society under a transformative agreement with JISC.

## Abbreviations

CAD: coronary artery disease
ECM: extracellular matrix
GRNs: gene regulatory networks
IEL: internal elastic lamina
SCA1: stem cell antigen-1
VSMCs: vascular smooth muscle cells
scRNA-seq: single-cell RNA-sequencing
